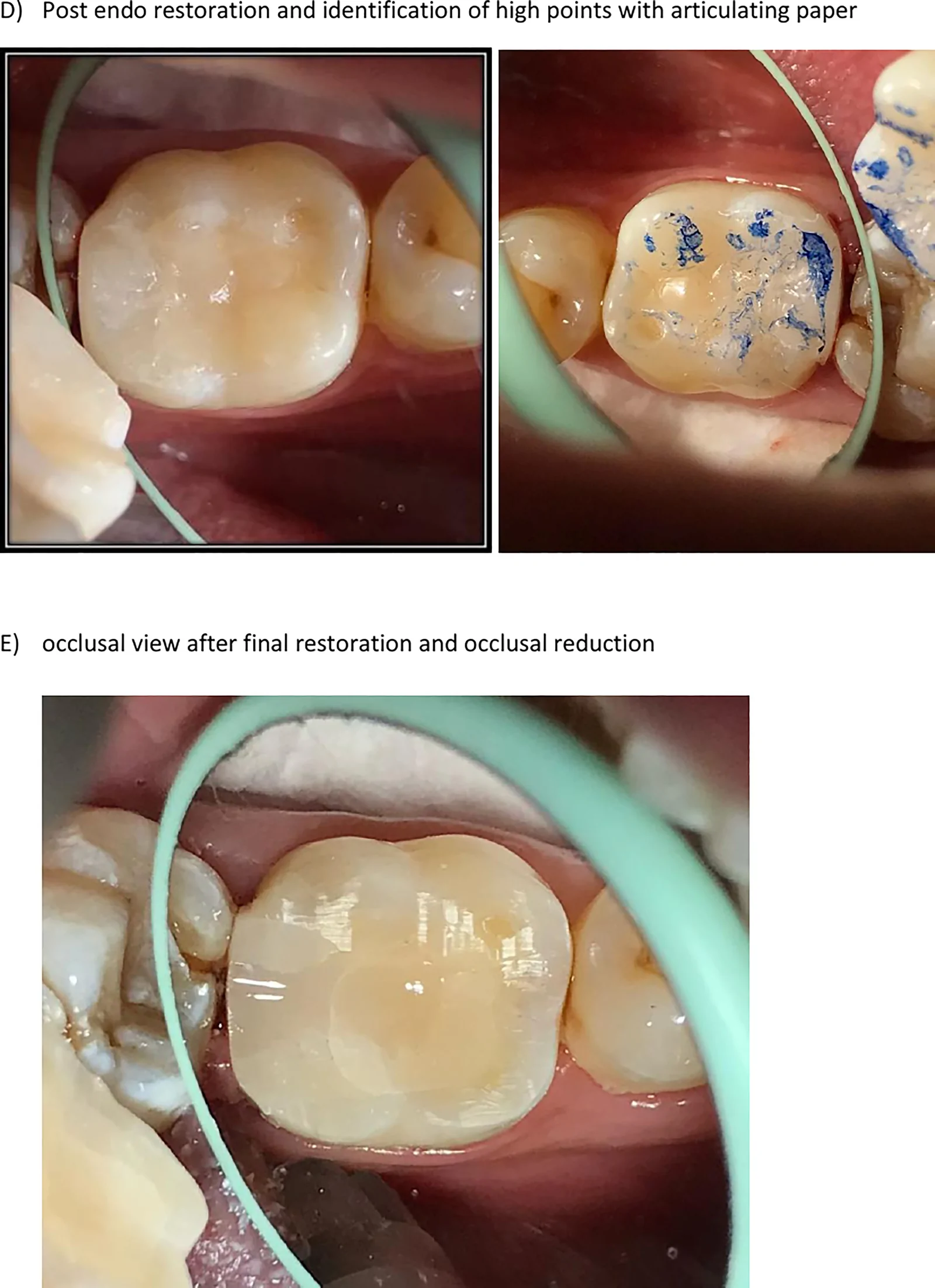# Effect of occlusal reduction on post-endodontic pain in teeth with irreversible pulpitis and symptomatic apical periodontitis: A randomized controlled trial

**DOI:** 10.1016/j.jobcr.2026.101535

**Published:** 2026-09-12

**Authors:** V. Yadav, M.I. Ansari, S. Katyal, B. Ahuja, S. Miglani

**Affiliations:** a Department of Dentistry, All India Institute of Medical Sciences, Bibinagar, India; b Faculty of Dentistry, Jamia Millia Islamia, New Delhi, 110025, India; c Deptt. of Pediatric Dentistry, K.D Dental college, Mathura, Uttar Pradesh, India

**Keywords:** Pain measurement, Postoperative pain, Pulpitis, Apical Periodontitis, Root canal treatment

## Abstract

**Background:**

This study evaluated the effect of occlusal reduction (OR) on post-endodontic pain intensity in individuals with carious molars diagnosed as irreversible pulpitis and symptomatic apical periodontitis.

**Methods:**

This randomized controlled trial included 200 participants (aged 18-44 years), who were randomly allocated to two groups. Preoperative pain score (T0) was recorded on a visual analogue scale (VAS). Group A received occlusal reduction, whereas Group B maintained intact occlusal surfaces. Endodontic treatment was performed over two visits by two experienced endodontists following standard root canal treatment protocols. Postoperative pain was subjectively recorded by using the VAS after the first [(T1 = 6h), (T2 = 12h), (T3 = 24h), (T4 = 48h), (T5 = 72h)] and second visits [(T6 = 6h), (T7 = 24h), (T8 = 48h)]. Statistical analysis was performed using the Mann–Whitney U, Wilcoxon signed-rank, and Friedman tests.

**Results:**

A total of 180 participants were assessed; pain scores at baseline were comparable between Group A and Group B (median: 8 [IQR: 3] (p = 0.900). Intergroup comparison demonstrated significantly lower pain scores in Group A than in Group B at **T1** (p = 0.021), **T2** (p = 0.033), and **T3** (p = 0.015). However, no statistically significant differences were observed at baseline or from **T4 to T8** (p > 0.05). By T4, the median pain score had decreased to zero in both groups and remained negligible throughout the subsequent follow-up period.

**Conclusion:**

Occlusal reduction significantly provides immediate pain relief up to 24 h after the first visit of root canal treatment in patients presenting with severe pain due to irreversible pulpitis and symptomatic apical periodontitis.

## Teaser

**Key message**- The result of this study supports the evidence for immediate occlusal reduction after the first visit of root canal treatment if there is severe preoperative pain due to irreversible pulpitis and symptomatic apical periodontitis in a tooth, irrespective of gender.

## 1 Introduction

Pain or discomfort after root canal treatment (RCT) is known as post-endodontic pain and is a common concern for patients and clinicians.^1^ Its incidence is approximately 3-58% following endodontic therapy, independent of the pulp status; however, a higher prevalence of 50-60% has been documented in individuals exhibiting peri-radicular pathologies.^2^ The manifestation of postoperative discomfort may be perceived by patients as an inadequacy on the part of the dental practitioner and can drastically affect the patient's quality of life, requiring prompt attention.^3^ Consequently, this phenomenon is the subject of increasing research interest, reflecting the need for a deeper understanding of the factors influencing post-endodontic pain and strategies to alleviate its intensity.^4^

Various strategies have been reported to reduce post-endodontic pain, including the pre-emptive or post-operative administration of analgesics, long-acting anaesthetics, low-level laser therapy, newer instruments and materials, and techniques such as occlusal reduction (OR). 4, 5, 6, 7, 8 Numerous studies have assessed the impact of OR on postoperative discomfort following root canal therapy. A few studies have reported that OR is beneficial in alleviating post-endodontic pain. 9, 10, 11, 12, 13 However, there is disagreement regarding the timing after the endodontic procedure at which this benefit is observed, with some reports finding no benefit.^14^, ^15^, ^16^, ^17^, ^18^, ^19^ This could be attributed to disparities in research design and methodology, including the pulpal/periapical condition, tooth type or position, the instrumentation technique employed, and the operator's expertise or specialization (Endodontist) across the studies.^20^, ^21^, ^22^ Consequently, there is a pressing need for meticulously designed clinical trials with larger cohorts to strengthen the existing evidence base, minimize heterogeneity and bias, and resolve the dilemma for practitioners regarding the appropriateness of OR after an RCT.

The investigation aims to determine whether occlusal reduction provides post-endodontic pain relief for patients with moderate or severe preoperative pain due to irreversible pulpitis and symptomatic apical periodontitis of mandibular molars, compared with no occlusal reduction.

## 2 Materials and methods

### 2.1 Study design and setting

This study was a prospective randomized clinical trial (two parallel arms, single-blind) in accordance with the Consolidated Standards of Reporting Trials (CONSORT) guidelines (Fig. 1).^13^ Institutional ethics committee approval was obtained (Proposal No. 26/6/283/JMI/IEC/2020, dated 03/07/2020) and the trial was registered with the Clinical Trials Registry of India (Reference No. CTRI/2021/02/031598, accessible at https://ctri.nic.in/). Written informed consent was obtained from the participants.^13^ The sample size was estimated from the study by Arslan et al. (2016),^18^ based on differences in mean postoperative pain scores at Day 3 (control: 11.27 ± 18.89; RCT intervention: 1.70 ± 4.15). Using OpenEpi (Version 3) with a two-sided α of 0.05, 90% power, and an equal allocation ratio, the minimum required sample size was calculated to be 43 participants per group. To enhance the robustness of the study, improve precision, narrow the confidence intervals, and account for potential dropouts, the sample size was increased to 100 participants per group (total n = 200).

**Fig. 1:**
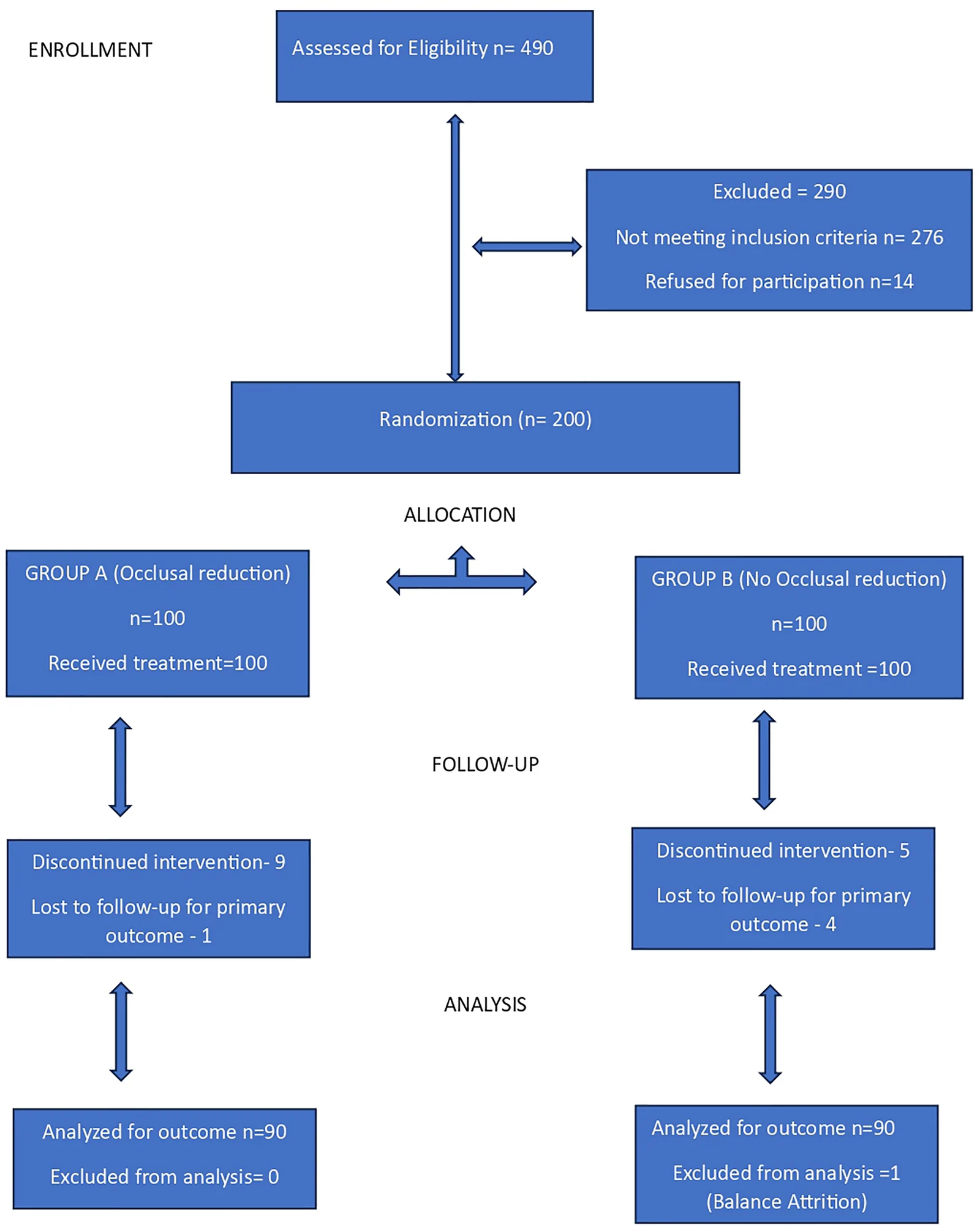
CONSORT Flow diagram depicting the progress through the phases of the randomized trial (that is, enrollment, intervention allocation, follow-up, and data analysis).

### 2.2 Participant selection

#### 2.2.1 Inclusion criteria

**Fig. 2:**
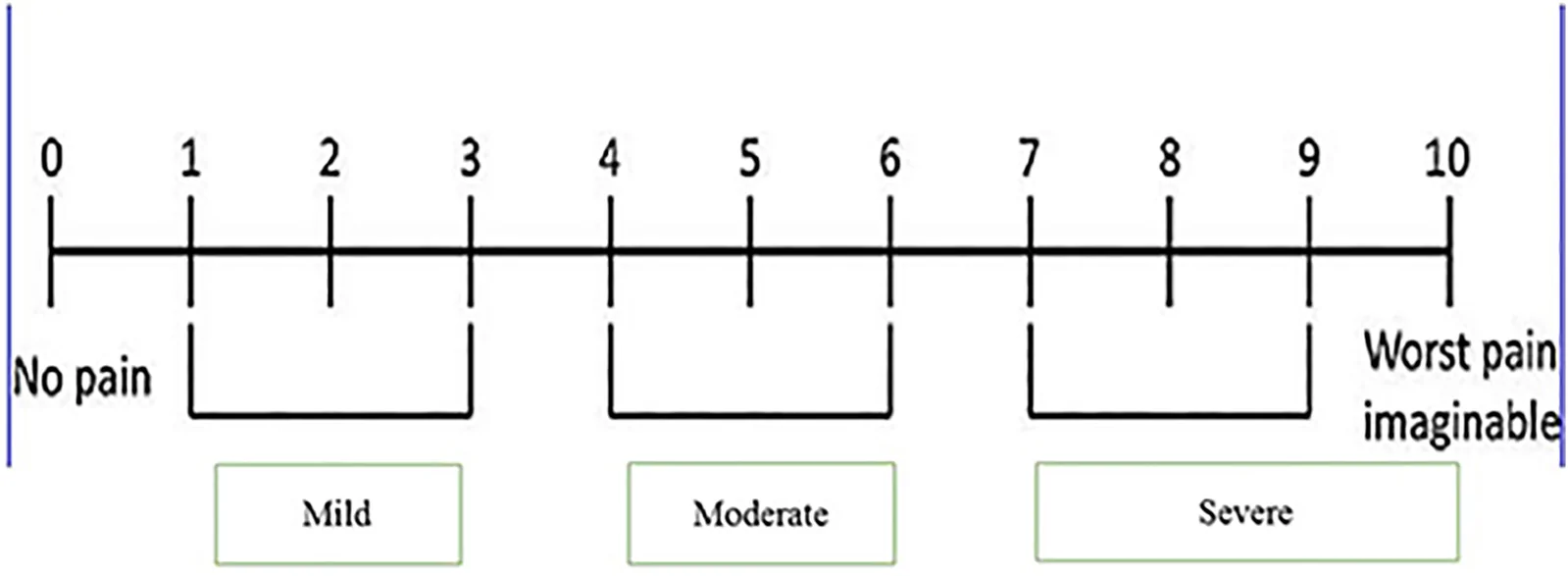
The VAS consists of a 10-cm line anchored by two extremes, "No pain" and "Worst imaginable pain". The patients were asked to choose the mark that represented their level of pain. Pain level will be assigned to one of the following categorical groups: None (0); Mild (1-3); Moderate (4-6); Severe (7-10). The scale was explained visually, verbally, and numerically to facilitate participants' use of it.

Healthy young adult participants (ASA I), aged 18–44 years, presenting with moderate to severe preoperative pain (T0) on the visual analogue scale (VAS) in carious mandibular molars with symptomatic irreversible pulpitis and apical periodontitis, were included in the study (Fig. 2).

The diagnosis was determined by integrating patient history, clinical findings, and radiographic assessments. The pain may be spontaneous or linger after exposure to cold or hot stimuli. Essential diagnostic criteria included a positive pulp sensitivity test (EPT and cold test), tenderness to percussion, lack of pain upon palpation, and the presence of mild periodontal ligament space widening without periapical radiolucency radiographically.

#### 2.2.2 Exclusion criteria

Participants with systemic disease or immunological disorders, pregnant women, those who took any medications (analgesics, antibiotics) within 24 h preoperatively, or a history of allergy to any medication or material used in the study; teeth without contact or with premature contact with an occluding tooth; teeth with more than two walls missing; teeth showing periodontitis (mobility > grade I or pocket depth > 5 mm); multiple missing teeth; parafunctional habits (bruxism); radiographic evidence of periapical pathology; or severely curved or calcified canal were excluded from the study.

### 2.3 Randomization and blinding

This was a single-blind study in which participants were unaware of their assigned treatment group. Participants were assigned to the intervention group (Group A: RCT with occlusion reduction) or the control group (Group B: RCT without occlusion reduction) in a 1:1 ratio, using a computer-generated randomization sequence (Random Allocation Software v1.0). The operator was not blinded to the assigned group because the treatment was apparent, and the patients were informed about both procedures, but were not aware of their allocation to a particular group. To maintain participant blinding, a bur was moved over the teeth without making contact (superficially) with the occlusal surface in Group B participants. The outcome assessor was blinded to the assigned group throughout the study.

### 2.4 Endodontic procedure

Experienced endodontists, trained in rotary endodontics, performed the procedure in accordance with the standard operating protocol and were supervised by a senior professor.

#### 2.4.1 First visit

Upon the establishment of pulpal anaesthesia and rubber dam isolation, the access cavity was prepared with a round carbide bur followed by an Endo-Z bur (Dentsply Maillefer, Ballaigues, Switzerland).^11^ Standard RCT crown-down protocol was followed. Stainless steel hand files (M-access, Dentsply) and Ni-Ti rotary files (Protaper Gold, Dentsply) were used.^12^^,^^13^ Following each instrumentation round, 1.5 mL of 2.5% sodium hypochlorite was used to cleanse the canal walls with ultrasonic activation.^13^ After drying of canals with paper points (25–30/2%), the access cavity was temporarily sealed with cotton and Cavit (3M ESPE, Seefeld, Germany)0.^11^, ^12^, ^13^.

Intact occlusion was assessed by placing articulating paper between the air-dried teeth in centric relation and examining the marks. For Group A, occlusal contacts were reduced on both the cusps (functional and non-functional) and the marginal ridges by 0.5-1 mm with a diamond wheel bur until satisfactory, as confirmed with 40-μm articulating paper in both centric and excursive relations (Arti-Check®, Bausch GmbH, Cologne, Germany) (Supplementary Figure).^9^ Normal occlusal contacts (without reduction) were left in Group B. It was ensured that Group A had no occlusal contact points and that Group B had normal occlusal contacts with no high points.

#### 2.4.2 Second visit

On the fourth day after the first visit, the patients were recalled, the canals were cleaned with 2.5% sodium hypochlorite and 17% EDTA, working length was re-established by sequentially using hand/rotary instruments, followed by obturation of the canals using the appropriate gutta-percha (Dentsply Pvt. Ltd, India) coated with sealer (AH plus, Dentsply, India) using warm vertical compaction technique. The final restoration with direct composite resin was done.^11^ (Filtek Z350XT,3M, India)

At the end of the first and second visits, all participants received counselling, and a placebo capsule, described as a mild analgesic, was provided for moderate pain. For severe pain, the patient was instructed to contact the operator or visit the hospital.

### 2.5 Outcome measures

Participants were instructed to record postoperative pain using the VAS chart at 6 h (T1), 12 h (T2), 24 h (T3), 48 h (T4), and 72 h (T5) after the first visit, and at 6 h (T6), 24 h (T7), and 48 h (T8) after the second visit. Telephonic reminder calls and text messages were used to ensure participant compliance throughout the trial. Non-compliance was recorded if a patient failed to attend a second visit or to record VAS scores at a given time interval.

### 2.6 Statistical analysis

Data were analyzed using SPSS Version 25.0 (IBM, Armonk, NY: IBM Corp., 2017). Normality was assessed using the Shapiro–Wilk test. Intergroup comparisons were performed using the Mann-Whitney *U* test, and Intra-group comparisons across multiple time points were done using the Wilcoxon signed-rank test and Friedman's test. A p-value of ≤ 0.05 was considered statistically significant.

## 3 Results

181 participants (90.5%) provided complete VAS scores from T1 to T8, indicating compliance, while 19 were lost due to missed visits or noncompliance. Consequently, 180 patients (73 males and 107 females; aged 18-44 years) were included in the final assessment, out of the original 200 participants (1 patient was excluded to balance attrition across groups). The mean age for both groups was comparable (28.9 ± 7.0 years for Group A and 30.7 ± 7.0 years for Group B) (Table 1). Although the study was initially designed as an intention-to-treat trial, participants with incomplete VAS data were excluded from the final analysis to maintain data completeness and reliability.

**Table 1:** Descriptive statistics of participants included in both groups.

Variables	Group A	Group B	p-value*
**Age (in Years)**			
Mean ± SD	28.9 ± 7.0	30.77 ± 7.0	0.08
Range	(17, 43)	(19, 46)	
**Gender [n (%)]**			
Male	35 (38.8%)	38 (42.3%)	0.20
Female	55 (61.2%)	52 (57.7%)	
**Preoperative VAS Score Category**			
Moderate (score 4-6)	28 (31.11%)	27 (30.0%)	0.8
Severe (score 7-10)	62 (68.89%)	63 (70.0%)	

*Independent *t*-test; significance p < 0.05.

The intergroup comparison showed significantly lower pain (VAS) scores in Group A (with occlusal reduction) from 6 to 24 h (T1, T2, T3) after the first visit for the RCT. There was no significant difference in pain intensity between the groups after 24 h from the first visit (i.e., at T4 and T5) and after the 2nd visit (T6-T8) (Table 2). The intragroup comparison showed a significant reduction in pain immediately after the first visit of root canal treatment in both groups (with and without reduction).

**Table 2:** Comparison of pain intensity (VAS) between both groups at various time points.

Time points	Group A				Group B				p-value ab
	Median	IQR	Min	Max	Median	IQR	Min	Max	
**T0**	8^A,a^	3	5	10	8^A,a^	3	5	10	0.9
**T1**	3**^A,b^**	3	0	8	4^B,b^	4	0	10	0.021*
**T2**	1^A,b^	2	0	6	1.5^B,b^	3	0	7	0.033*
**T3**	0^A,b^	1	0	4	0.5^B,b^	2	0	6	0.015*
**T4**	0^A,b^	0	0	1	0^A,b^	0	0	4	0.45
**T5**	0^A^	0	0	1	0^A^	0	0	1	0.91
**T6**	0^A^	0	0	2	0^A^	0	0	3	0.581
**T7**	0^A^	0	0	1	0^A^	0	0	1	0.71
**T8**	0^A^	0	0	0	0^A^	0	0	0	-
**p-value**^c^	<0.01*				<0.01*				

IQR: Interquartile range; Min: Minimum; Max: Maximum; SD: Standard deviation.

a T-test comparing two independent groups in a row; Time points: Pre-treatment (T0); After first visit: At 6h (T1); 12h (T2); 24h (T3); 48h (T4); 72h (T5); After second visit: At 6h (T6); 24h (T7); 48h (T8).

b Mann-Whitney *U* test for inter-group comparison at different time points (In a row, different capital superscripts represent significant difference).

c Freidman's test for intra-group comparison at all time-points, *p-value <0.05. (In a column, different small letter superscripts represent a significant difference).

In the categorical analysis (severe vs. moderate), the difference in pain intensity was significant in the severe pain category up to 24 h postoperatively, but not in the moderate pain category (Table 3).

**Table 3:** Intergroup Comparison of Pain intensity in moderate and severe category patients.

Time points	Severe Pain category (Median VAS score)			Moderate Pain category (Median VAS score)		
	Group A	Group B	p-value^a^	Group A	Group B	p-value^a^
**T0**	9^A^	9^A^	NS	6^A^	5.5^A^	NS
**T1**	3^B^	4^B^	0.001*	0^A^	0.5^A^	0.796
**T2**	2^B^	2^B^	0.002*	0^A^	0^A^	0.326
**T3**	1^B^	1.5^B^	0.002*	0^A^	0^A^	-
**T4**	0^B^	0^B^	0.338	0^A^	0^A^	-
**T5**	0^B^	0^B^	0.952	0^A^	0^A^	-

Time points: Pre-treatment (T0); After first visit: At 6h (T1); 12h (T2); 24h (T3); 48h (T4); 72h (T5); After second visit: At 6h (T6); 24h (T7); 48h (T8).

a Mann-Whitney *U* test; NS: non-significant; significant p < 0.05. (In a row, different capital superscripts represent a significant difference).

Binary logistic regression analysis showed that post-endodontic pain was significantly associated with preoperative pain [OR = 1.2, 95% CI 1.0–1.3], indicating that patients with severe preoperative pain had 1.2 times higher odds of experiencing post-treatment pain. In contrast, gender was not associated with post-endodontic pain [OR = 0.95, 95% CI: 0.74–1.0, p = 0.21], suggesting no significant difference in pain between males and females (Supplementary Table 1).

A total of 54/180 participants received placebo capsules after the 1st visit (28/90 in Group A, 26/90 in Group B), and 10/180 received placebo capsules after the 2nd visit (6/90 in Group A, 4/90 in Group B). There was no statistically significant difference between the groups for placebo medication intake. No patient reported to the hospital due to extreme pain after the 1st and 2nd visits.

## 4 Discussion

This study evaluated the effectiveness of occlusal reduction during root canal treatment for relieving postoperative pain. The findings indicate that pain intensity decreased significantly immediately after occlusal reduction in patients with severe preoperative pain, compared with those with moderate pain (up to T3). Furthermore, postoperative discomfort subsided completely within 72 h after the first appointment in both groups. These results suggest that occlusal adjustment primarily benefits the early postoperative period, while the subsequent resolution of pain likely reflects the natural subsidence of inflammation following endodontic therapy. There was no difference in postoperative pain between the two groups after the second visit at any time point (T6-T8).

Endodontic treatment of vital mandibular molars with pre-existing apical periodontitis can lead to severe postoperative pain, attributed to factors such as the individual's pain threshold, genetic and environmental influences (tooth type and location), pulpal condition, preoperative tenderness, preoperative periapical condition, extrusion of microbes, infected debris and irrigation solutions into the periapical tissue, over-instrumentation, and pressure on the apical periodontal ligament (PDL) nerve fibers from premature or permanent occlusal contacts.^4^^,^^5^^,^^23^ OR has been proposed as a mechanical strategy for reducing postoperative pain. The primary justification in minimizing post-endodontic pain is its ability to alleviate “mechanical allodynia” by preventing repeated stimulation of already sensitized pain receptors in the periodontal ligament due to periapical inflammation.^24^ The result of this study supports the evidence that teeth with severe preoperative pain have greater susceptibility to mechanical stimulation of the periodontal ligament, which is relieved by OR.

Several clinical studies have compared the effectiveness of OR in reducing post-RCT pain in symptomatic vital or non-vital teeth, with or without apical periodontitis, and have reported contrasting findings. 9, 10, 11, 12, 13, 14, 15, 16, 17, 18, 19 Previous studies have suggested that OR can help to reduce the incidence of post-procedural endodontic pain.^9^, ^10^, ^11^, ^12^, ^13^ Rosenberg et al. (1998) reported that occlusal trimming reduces postoperative pain after 48 h in teeth with vital pulp, particularly in cases with preoperative pain and sensitivity to percussion, but with the absence of peri-radicular radiolucency.^9^ Emara et al. (2019) and Y.E. Ahmed et al. (2020) found that occlusal reduction successfully alleviated pain within 12-24 h after the first RCT visit, similar to the results observed in this study after the 1st visit.^12^^,^^13^ Only two studies evaluated the pain intensity after the 2nd visit (post-obturation), where Emara et al. reported pain reduction following occlusal reduction after the 2nd visit, whereas Y.E. Ahmed et al. (2020) found no benefit of OR in decreasing pain intensity after the 2nd visit post-obturation.^12^^,^^13^ A placebo analgesic was given to differentiate the participant who takes analgesics due to fear. No participant reported for an emergency inter-appointment visit. The result of this study is consistent with the existing literature described above.^13^

Other studies, including Parirokh et al., 2013, Asghar et al. (2014), Arslan et al. (2017), Raza et al. (2016), Manigandan et al. (2024) and systematic reviews, reported no benefit of occlusal reduction in reducing postoperative pain up to 6 days after an endodontic procedure.^14^, ^15^, ^16^, ^17^, ^18^, ^19^, ^20^, ^21^, ^22^ However, all these studies have a high risk of bias and differ in sample population, treatment protocol, methodology, operator's skill, disease condition, and type of tooth.^20^, ^21^, ^22^.

OR is an irreversible procedure that removes sound tooth structure and may have adverse structural and biological consequences if not restored properly with definitive restoration such as an onlay, overlay or crown. Therefore, the decision to perform OR as a strategy for post-endodontic pain relief should be taken cautiously. This study supports that in severe preoperative pain conditions OR can help in immediate pain relief. However, its use should be considered selective and clinically justified rather than adopted routinely for all endodontically treated teeth.

The strengths of this study include its efforts to minimize potential confounding factors that could exacerbate or reduce postoperative pain, ensuring a valid comparison between the groups. Mandibular teeth are associated with greater postoperative pain than maxillary teeth; thus, with strict inclusion and exclusion criteria, only mandibular molars were included to establish baseline similarities.^25^ Pulpal diagnosis was validated using electric pulp testing and cold testing.^14^ Teeth with periapical lesions were excluded because they tend to exhibit more severe post-treatment pain following endodontic therapy.^26^ Teeth without an antagonistic tooth or with premature contacts were also excluded, as the teeth under study needed to have normal contacts to simulate ideal clinical conditions.^24^ In the present study, procedures were standardized based on previous studies.^12^^,^^13^ The endodontic procedure was completed in two visits by trained endodontists, and a 2.5% sodium hypochlorite solution was used for irrigation with ultrasonic activation during the procedure (Standardized calibrated operator/protocol).^27^ No intracanal medicament was used between visits, in accordance with established protocols.^28^^,^^29^ It was ensured that the temporary restoration after the first visit did not have any high points that could further accentuate post-endodontic pain by causing pressure on the PDL due to premature contact.^24^ The assessment of pain using VAS, as adopted in the present study, aligns with the methodology used in most earlier studies 13, 14, 15, 16 as it is considered one of the simplest and most reliable methods for assessing pain.^30^ Pain was recorded over a longer period as post-endodontic pain may last up to 1 week after treatment.^2^^,^^13^ Blinding was ensured to prevent potential assessment bias.

This study's limitations include use of self-reported pain scale which may introduce measurement variability. Endodontic treatment was done in two-visit rather than a single-visit procedure and excluding non-vital teeth, which restricts the generalizability of the findings. This study has limited external validity due to single center involvement. Consequently, additional high-quality multi-centric research is needed to assess the role of occlusal reduction in reducing post-treatment pain after single-visit endodontic procedures across diverse pulpal and peri-radicular conditions in a larger population.

## 5 Conclusion

The findings of the present study suggest a significant benefit of occlusal reduction in alleviating immediate post-endodontic pain up to 24 h, especially when the preoperative pain score is severe in a vital tooth with percussion sensitivity. However, in case of moderate preoperative pain, alternative pain management strategies (analgesic intake) can be opted.

## Informed consent

Written informed consent was obtained from the participants.

## Ethical statement

Institutional ethics committee approval was obtained (Proposal No. 26/6/283/JMI/IEC/2020, dated 03/07/2020). Trial was registered with the Clinical Trials Registry of India (Reference No. CTRI/2021/02/031598, accessible at https://ctri.nic.in/).

## Human and animal statement

The study protocol was approved by Institutional Ethics Committee with Proposal No. 26/6/283/JMI/IEC/2020, dated 03/07/2020 (Jamia Millia Islamia University). All procedures involving human participants were performed in accordance with the ethical standards of the institutional research committee and the Helsinki Declaration. The trial was registered with the Clinical Trials Registry of India (Reference No. CTRI/2021/02/031598, accessible at https://ctri.nic.in/). Written informed consent was obtained from the participants.

## Source(s) of support

The study was funded by council of scientific and industrial research institute (CSIR, HRDG complex, New Delhi, INDIA) (Grant no. B-12712). Role of funding agency was to provide salary to senior research associate and contingency cost of instruments used to carry out the intervention.

## Declaration of competing interest

The authors declare that they have no known competing financial interests or personal relationships that could have appeared to influence the work reported in this paper.

## Figures and Tables

**figs1:**
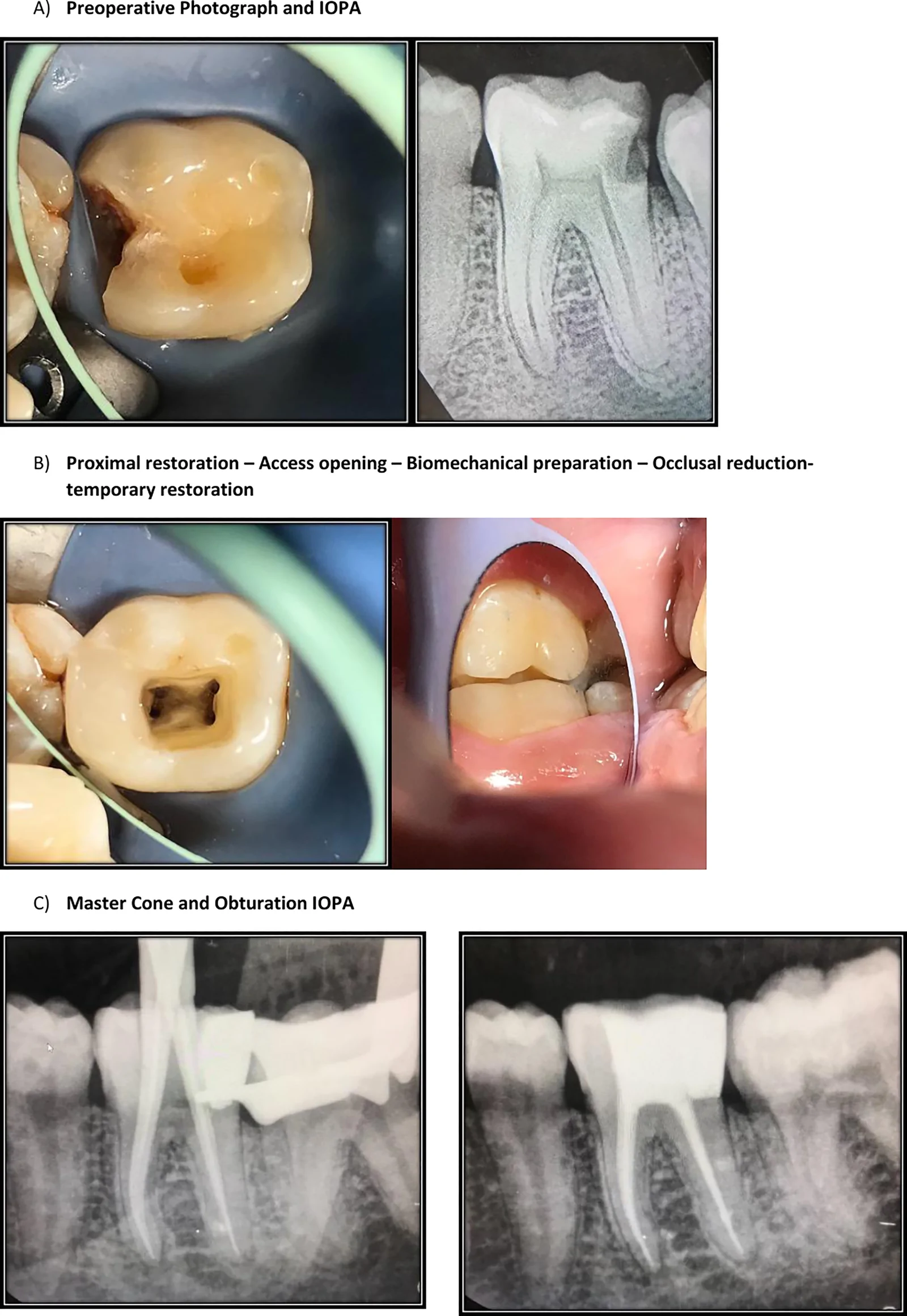


**figs2:**