# Supplementary material for: Effect of occlusal reduction on post-endodontic pain in teeth with irreversible pulpitis and symptomatic apical periodontitis: A randomized controlled trial
Source: J Oral Biol Craniofac Res. 2026 Sep 12;16(6):101535. doi: 10.1016/j.jobcr.2026.101535 (PMC13587906; doi:10.1016/j.jobcr.2026.101535)
Supplement: Supplementary file 1 [file mmc1.docx]

**Supplementary Table 2: Binary logistic regression of the effect of variable factors on overall post-endodontic pain.**

| **Variables**  **(Test category/ Reference category)** | **S.E.** | **OR** | **95% CI** | | **p-value** |
| --- | --- | --- | --- | --- | --- |
|  |  |  | **Lower bound** | **Upper bound** |  |
| Gender (Male/Female) | 0.2 | 0.95 | 0.74 | 1.0 | 0.21 |
| Preoperative pain (Moderate/ Severe) | 0.1 | 1.2 | 1.3 | 1.0 | 0.05* |

*S.E.: Standard error; OR: Odds ratio; CI: Confidence interval; * indicates statistical significance p<0.05*
